# Supplementary material for: “You do need each member of the team to bring that next piece of the puzzle”: Allied health professionals’ experience of interprofessional complex care in hospital settings
Source: PLoS One. 2025 Mar 14;20(3):e0317799. doi: 10.1371/journal.pone.0317799 (PMC11908697; doi:10.1371/journal.pone.0317799)
Supplement: S1 File — (DOCX) [file pone.0317799.s001.docx]

**Interview Questions:**

| 1. **Learning about the person’s motivations, values, and what matters to them:** |
| --- |
| Could you start by telling me a bit about yourself please –   - 1. Could you describe for me what your role entails; what do you do in the hospital and where does your work take you?   2. Could you tell me a little bit about how you came to choose the profession you are in? Is this something you have always wanted to do, or has it been a journey of sorts?   3. What is it about this field of work that interests you?   4. How did you come to think about your role in the way that you do now? Were there experiences that were particularly formative? Can you talk about any of them?   5. Every day you are working with patients that have four or more chronic health conditions. In addition, they may have a range of other (social) problems also. What must you bring of yourself to this work to be effective in your role? |
| 1. **Understanding the context of care** |
| 1. Could you tell me about your workload and its complexity? 2. Describe the environment in which you work? 3. Are there things that get in the way of you doing your job as well as you would like to? Can you describe these? 4. How do the available systems and supports help you in your work? |
| 1. **Learning about how the person’s perspectives, values, and motivations inform their practice:** |
| 1. Describe for me how you would approach an initial encounter with a new patient? 2. How do you go about shaping a conversation with a patient? What is important to focus on in the conversation? 3. Are there aspects that take priority in your interactions with patients? What might be considered more peripheral, and how do you prioritise what matters most in your interactions over what is less important? 4. How do you gain the trust of the patient in your care? 5. What do you and the team around you provide the patient? 6. What does the process of interaction with a patient feel like for you? 7. What makes for an effective interaction as opposed to a less effective one? 8. Are some clinical settings better than others? How are they different. 9. What happens when rapport building falters – how do you manage that?      1. What are the limitations of time with your patients? Can you give an example? 2. How does the issue of limited time impact upon the quality of your interactions with patients? 3. How does limited time impact on your interactions with other team members? 4. How does patient behaviour effect the quality of your interactions? 5. Are there any other patient level factors that influence the quality of the clinical relationship that you form with them? 6. Can you describe for me a difficult or complex interaction that you have had with a patient? |
| 1. **Linking the interviewee’s motivations, values, and practices to their world view of chronic disease:** |
| We know that chronic disease is a large and complex problem both in Australia and globally; we know also that social determinants have a large role in this trend.   - 1. How do you see the role of the hospital in terms of the broader patient issues – such as unstable housing, domestic violence, disability, social disadvantage, addiction, or mental illness?   2. People with chronic disease may present frequently to the hospital.   What is that like for you, in your role, encountering the same patient returning to hospital for the same types of health-related problems?   - 1. Please describe what you believe to be the patient’s role in managing their health conditions? How do you engage with your patients to promote their well-being?   2. Describe a recent patient who presented with chronic health issues and complex needs. Please do not disclose identifying details. |
| 1. **Broad overview – closing questions** |
| - Overall - How would you describe the purpose of the modern hospital – what is its role and function? - If you were to sum up the purpose of your role in one or two sentences – what would you say? |
