# Supplementary material for: “You do need each member of the team to bring that next piece of the puzzle”: Allied health professionals’ experience of interprofessional complex care in hospital settings
Source: PLoS One. 2025 Mar 14;20(3):e0317799. doi: 10.1371/journal.pone.0317799 (PMC11908697; doi:10.1371/journal.pone.0317799)
Supplement: Attachment — Submitted filename: Response to reviewers letter.docx [file pone.0317799.s003.docx]

Dear Editors,

In addressing the reviewer’s comments:

Include captions at the end of the article:

- Captions have been included at the end of the manuscript to match figures which have been provided separately as supporting information files (as requested).

Ensure the manuscript follows the style guide

- The style guide has been followed to the best of our knowledge

Data availability not available for proprietary reasons – must have a key point of contact that is not an author, nor an individual.

- The authors have provided a link to deidentified data, therefore, access to the data does not require contact with an individual.

Amend the title of the article to match the online submission form

- The title has been amended to match the online submission form

Amend table two to make it more clear with lines that show column 2 is a continuation of the analysis in column 1.

- Table 2 has been amended as requested.

The length of interviews should be included.

- The length of interviews has been included in the data collection section

Figure 1 is blurry, provide a clearer picture of picture 1.

- Figure 1 has been updated to be clearer

Preference is to not have figures in the discussion section.

- The figures in the discussion section were deemed necessary to help illustrate how the data have been interpreted.

Amend the article to read as being more scientific

- We have reviewed thoroughly how we described the study’s conceptual framework and the methods used in data collection and analysis, and framed the discussion accordingly. We hope this meets the editors’ expectations. As this research was qualitative there were no statistical data to present. However, we are happy to make further changes should this be required.

We hope you find this submission improved and look forward to hearing from you,

Kind regards

Felice Borghmans

[felice.borghmans@monash.edu](mailto:felice.borghmans@monash.edu);

[feliceborghmans@gmail.com](mailto:feliceborghmans@gmail.com)
